# Supplementary material for: Innate-like self-reactive B cells infiltrate human renal allografts during transplant rejection
Source: Nat Commun. 2021 Jul 16;12:4372. doi: 10.1038/s41467-021-24615-6 (PMC8285506; doi:10.1038/s41467-021-24615-6)
Supplement: Supplementary file 1 — Supplementary Information [file 41467_2021_24615_MOESM1_ESM.pdf]

Supplementary Information for

“Innate-like, self-reactive B cells infiltrate rejecting human renal allografts”

Yuta Asano[1], Joe Daccache[2], Dharmendra Jain[3], Kichul Ko[1], Andrew Kinloch[1], Margaret Veselits[1], Donald Wolfgeher[4], Anthony Chang[5], Michelle Josephson[6], Patrick Cunningham[6], Anat Tambur[7], Aly A. Khan[5], Shiv Pillai[8], Anita S. Chong[3], Marcus R. Clark[1]*

[1]Section of Rheumatology and The Knapp Center for Lupus and Immunology Research, Department of Medicine, The University of Chicago, Chicago, IL, USA.

[2]Transplantation Research Center, Renal Division, Brigham and Women’s Hospital, Harvard Medical School, Boston, MA, USA.

[3]Section of Transplant, Department of Surgery, The University of Chicago, Chicago, IL, USA.

[4]Department of Molecular Genetics and Cell Biology, The University of Chicago, IL, USA.

[5]Department of Pathology, The University of Chicago, Chicago, IL, USA.

[6]Section of Nephrology, Department of Medicine, The University of Chicago, Chicago, IL, USA.

[7]Transplant Immunology Laboratory, Northwestern University, Chicago, IL, USA.

[8]Ragon Institute of Massachusetts General Hospital, Massachusetts Institute of Technology, and Department of Medicine, Harvard Medical School, Boston, MA, USA.

*Corresponding author. Email: mclark@uchicago.edu

Cohort		First									Second					
Patient		Kidney 1	Kidney 2	Kidney 3	Kidney 4	Kidney 5	Tonsil 1	Tonsil 2	Tonsil 3	Tonsil 4	Kidney 6	Kidney 7	Kidney 8	Tonsil 5	Tonsil 6	Tonsil 7
Gender		4 Females and 1 Male									3 Males					
Age		Mean = 50, s.d. = 15.6									Mean = 55, s.d. = 9.5					
Kidney survival (yr)		Mean = 11.3, s.d = 8.6									Mean = 9.2, s.d.= 11.1					
Disease		HT	IgAN	HT	RN	MCKD					HT, T2D	HT	T1D			
Diagnosis		Chronic AMR	Chronic AMR	Chronic active AMR	Chronic active AMR	Chronic AMR					Mixed / Active AMR	Active AMR	Chronic active AMR			
DSA		No	No	No	Yes	No					Yes	Yes	No			
	c4d staining, c4d	0	0	0	2	0					3	3	1			
	tubulitis, t	1	1	0	0	0					3	0	0			
	inflammation, i	2	1	1	1	0					2	0	1			
	intimal arteritis, v	0	0	0	0	0					1	0	0			
	glomerulitis, g	1	2	0	2	0					0	0	0			
Banff score	interstitial fibrosis, ci	2	2	3	1	1	NA				2	1	1	NA		
	tubular atrophy, ct	2	2	3	1	1					2	1	1			
	chr allgx gp, cg	1b	3	0	3	0					0	0	0			
	intimal thickening, cv	1	1	0	1	1					3	1	1			
	peritubular capillaritis, ptc	1	2	1	1	2					2	0	0			
	total infl, ti	3	3	3	3	1					2	1	2			
	i-IFTA	3	3	3	2	2					2	1	2			
Cr		1.9	3	1.4	2.4	1.8					11.1	1.4	2.7			
eGFR		36	19	37	29	30					5	51	25			
Proteinuria		2.5	2.7	7.2	1	0.13					0.96	0.09	1.4			
Latest followup		Cr 4.2 at 50 months post-biopsy	Cr 4.8 at 56 months post-biopsy	Cr 1.6 at 52 months post-biopsy	Cr 4.3 at 35 months post-biopsy; Deceased	Cr 1.6 at 12 months post-biopsy					Cr 10.5 at 34 months post-biopsy; On dialysis since 1 month post-biopsy	Cr 1.1 at 23 months post-biopsy	Cr 6.2 at 22 months post-biopsy; On dialysis since 15 months post-biopsy			
Cell infiltrates		T/B aggregates	Diffuse	Diffuse	Diffuse	Diffuse					T/B aggregates	T/B aggregates	T/B aggregates			
Sorted cells		96	336	96	192	192	48	48	96	96	192	96	96	96	96	96
Cells after QC		71	262	69	77	176	43	41	21	24	137	51	77	87	81	84

Abreviations...HT: Hypertension, IgAN: IgA nephropathy, RN: Reflux nephropathy, MCKD: Medullary cystic kidney disease, T1D/T2D: Type-1/2 diabetes, AMR: Antibody-mediated rejection, Cr: Serum creatinine, eGFR: Estimated glomerular filtration rate

Supplementary Table 1. Patient information.

For transplant patients, age, gender and kidney survival length are masked to protect patients' confidentiality. Tonsil donors are deidentified.

Ig class	Kidney 1	Kidney 2	Kidney 3	Kidney 4	Kidney 5	Tonsil 1	Tonsil 2	Tonsil 3	Tonsil 4	Kidney 6	Kidney 7	Kidney 8	Tonsil 5	Tonsil 6	Tonsil 7	Total
IGHM	27	165	31	68	111	33	20	15	10	13	19	33	37	21	43	646
IGHD	0	10	0	0	0	0	0	1	0	10	2	1	8	14	26	72
IGHA1	10	13	9	2	18	1	1	3	0	10	7	11	8	11	3	107
IGHA2	1	3	1	0	2	0	0	0	0	0	1	4	1	0	1	14
IGHG1	23	52	19	2	30	6	15	1	11	88	16	11	16	23	4	317
IGHG2	4	13	2	3	11	0	1	0	0	4	2	8	6	4	5	63
IGHG3	6	6	7	2	3	3	4	1	3	12	2	9	11	8	2	79
IGHG4	0	0	0	0	0	0	0	0	0	0	1	0	0	0	0	1
IGHE	0	0	0	0	1	0	0	0	0	0	1	0	0	0	0	2
Total	71	262	69	77	176	43	41	21	24	137	51	77	87	81	84	1301

Supplementary Table 2. Ig class distribution across patients.

Number of QC-passed cells grouped by Ig class are shown for each patient.

Group 1	Group 2	p.value	FDR
B1a.PC	B1a.Sp	0.039	0.29
B1a.PC	B.Fo.PC	0.033	0.29
B1a.PC	B.MZ.Sp	0.032	0.29
B1a.PC	B.T1.Sp	0.024	0.29
B1a.PC	B.T2.Sp	0.037	0.29
B1a.PC	B.T3.Sp	0.37	0.37
B1a.PC	B.GC.Sp	0.00013	0.002
B1a.PC	B.Fo.Sp	0.043	0.29
B1b.PC	B1a.Sp	0.02	0.27
B1b.PC	B.Fo.PC	0.025	0.29
B1b.PC	B.MZ.Sp	0.026	0.29
B1b.PC	B.T1.Sp	0.018	0.25
B1b.PC	B.T2.Sp	0.024	0.29
B1b.PC	B.T3.Sp	0.18	0.36
B1b.PC	B.GC.Sp	0.00011	0.0018
B1b.PC	B.Fo.Sp	0.035	0.29

Supplementary Table 3. Results of t-test and FDR for Figure 3G.

Peritoneal cavity B1a and B1b cells were compared by t-test with the other B cell populations in Immgen for DEG cluster 3 gene expression scores. n = 6 for B1a.PC, and n = 3 for the other populations.

	1C	11AV	65QKR	66K	95L	103L	113YD	116S	152E	152RE	163T	184H	193PV	194V
C*06:02	x	x	x	x	x	x	x	x	x	x	x	x	x	x
C*07:02	x	x	x	x	x	x	x	x			x			
C*14:02	x		x	x	x	x	x	x	x	x	x	x	x	x
C*02:02	x	x	x	x	x	x	x	x	x	x		x	x	x
C*16:01	x	x	x	x	x	x	x	x			x	x		x
C*04:01			x	x	x	x			x	x	x	x	x	x
C*18:02	x	x	x	x	x	x			x	x	x	x	x	x
C*05:01	x	x	x	x	x	x			x	x	x	x	x	x
C*01:02	x		x	x	x	x	x		x	x	x	x	x	x
C*12:03	x	x	x	x	x	x	x	x	x	x	x	x	x	x

Supplementary Table 4. Eplets shared among top-10 hits for 3-1E12

Those shared among all the top-10 alleles (65QKR and 103L) were highlighted.

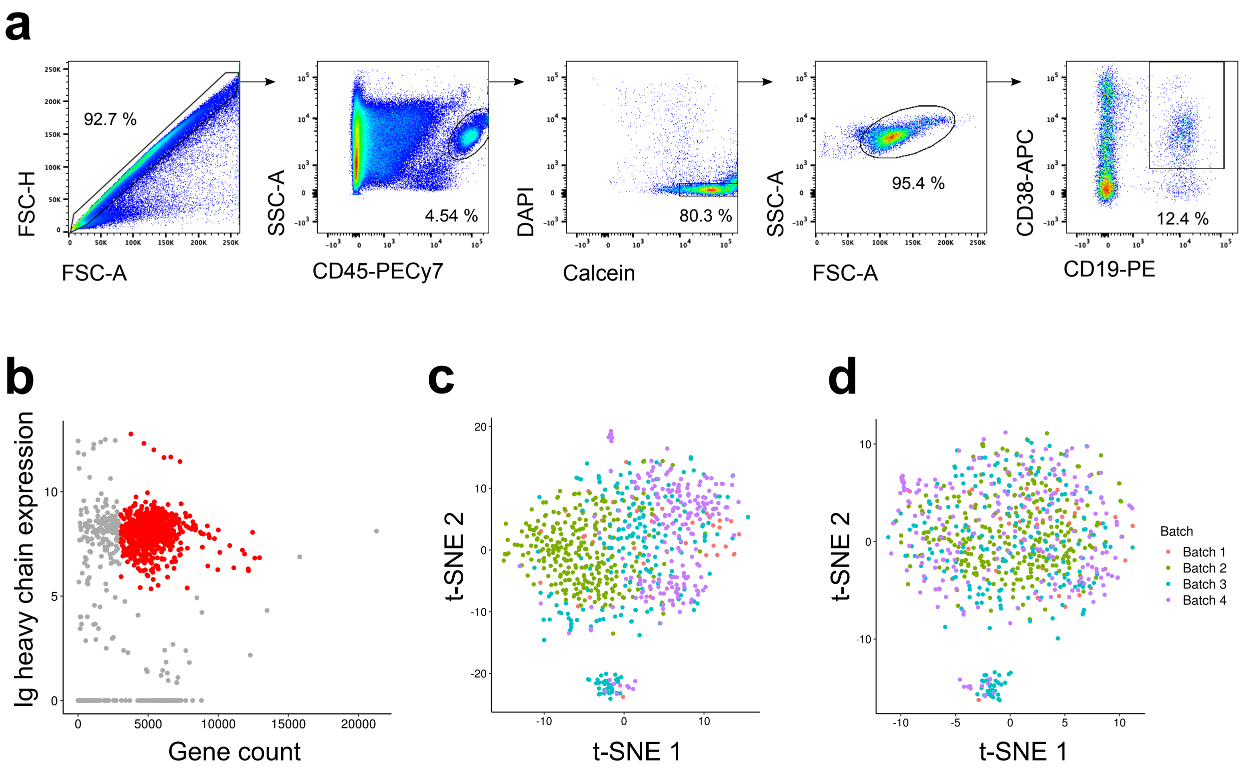

Supplementary Figure 1. QC and normalization with ERCC spike-in to dissolve batch-dependent clusters.

(**a**) A full gating strategy for intrarenal B cells in Figure 1A. (**b**) A scatter plot showing detected gene count and Ig heavy chain gene expression on the x and y axis respectively. Cells which passed QC are colored in red. (**c** and **d**) t-SNE plots colored by experimental batches before (c) and after (d) batch normalization with ERCC spike-in and RUVSeq.

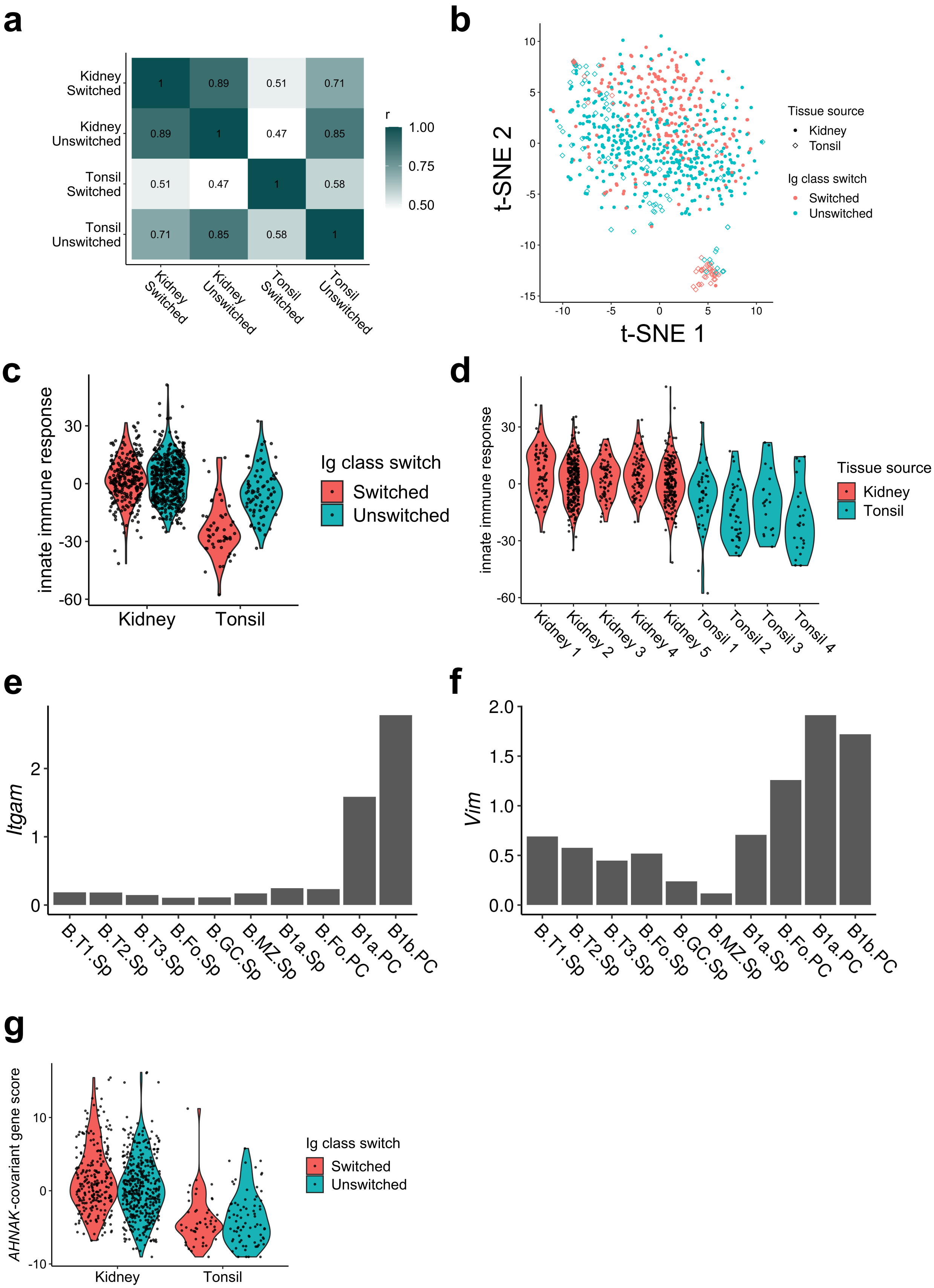
a
b
c
d
e
f
g
Kidney Switched
Kidney Unswitched
Tonsil Switched
Tonsil Unswitched
r
1.00
0.75
0.50
1
0.89
0.51
0.71
0.89
1
0.47
0.85
0.51
0.47
1
0.58
0.71
0.85
0.58
1
t-SNE 2
t-SNE 1
Tissue source
Kidney
Tonsil
Ig class switch
Switched
Unswitched
innate immune response
Kidney
Tonsil
Ig class switch
Switched
Unswitched
innate immune response
Kidney 1
Kidney 2
Kidney 3
Kidney 4
Kidney 5
Tonsil 1
Tonsil 2
Tonsil 3
Tonsil 4
Tissue source
Kidney
Tonsil
Itgam
B.T1.Sp
B.T2.Sp
B.T3.Sp
B.Fo.Sp
B.GC.Sp
B.MZ.Sp
B1a.Sp
B.Fo.PC
B1a.PC
B1b.PC
Vim
B.T1.Sp
B.T2.Sp
B.T3.Sp
B.Fo.Sp
B.GC.Sp
B.MZ.Sp
B1a.Sp
B.Fo.PC
B1a.PC
B1b.PC
AHNAK-covariant gene score
Kidney
Tonsil
Ig class switch
Switched
Unswitched

Supplementary Figure 2. Innate immune and B1-like gene expression in intrarenal B cells.

(**a**) Pearson correlation coefficients between B cell populations for expression of 1,000 most variant genes. (**b**) A t-SNE plot as in Figure 2A, in which Ig constant region genes were removed from the data. (**c** and **d**) A sum of scaled expression values of genes tagged to "innate immune response" GO term in cluster 2-3. Data were grouped by tissue sources (c) or patients (d). (**e** and **f**) Expression of *Itgam* (e) and *Vim* (f) in the mouse B cell subsets. (**g**) Gene expression scores for 13 *AHNAK*-covariant genes in DEG cluster 3 excluding *AHNAK*.

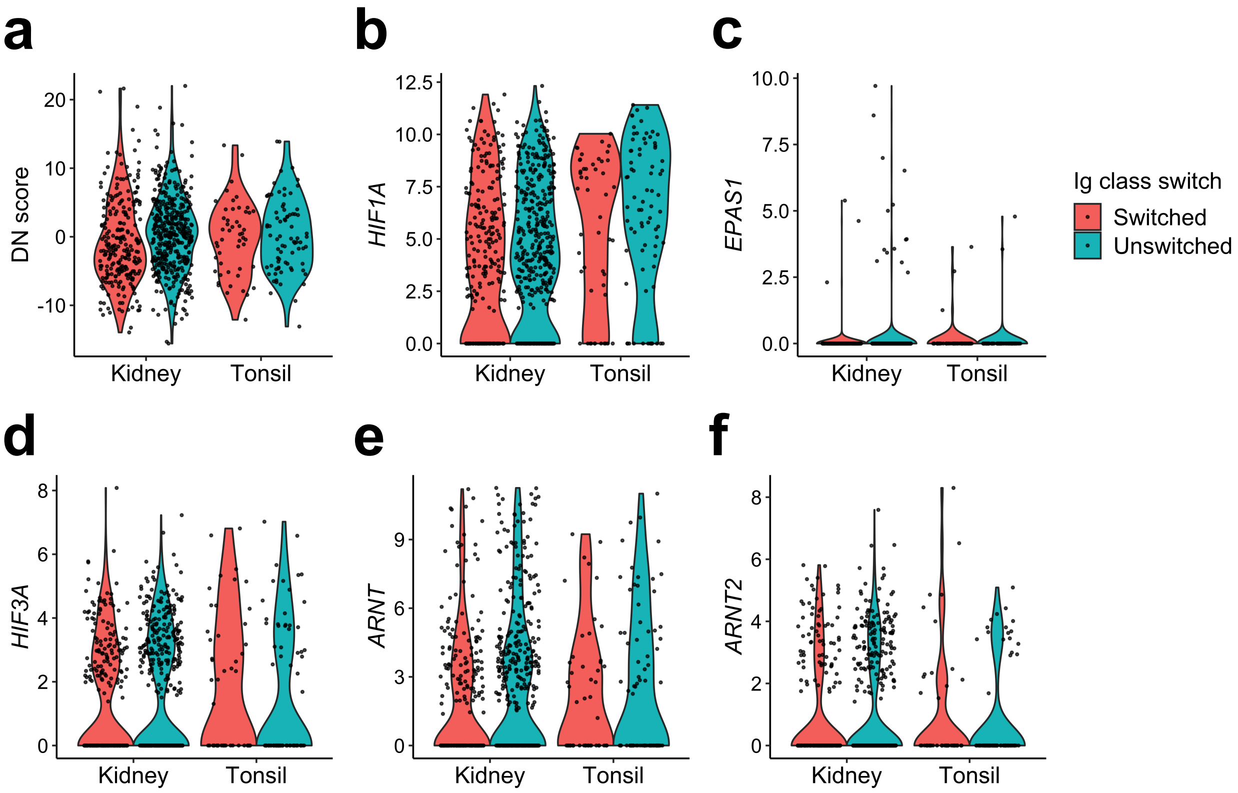

Supplementary Figure 3. Intrarenal B cells had no association with ABC and HIF signatures. (**a**-**f**) Violin plots showing the gene expression of ABC-associated genes (a), *HIF1A* (b), *HIF2A* (c), *HIF3A* (d), *HIF1B* (e), or *HIF2B* (f). Cells were grouped by their tissue source and Ig class-switch state.

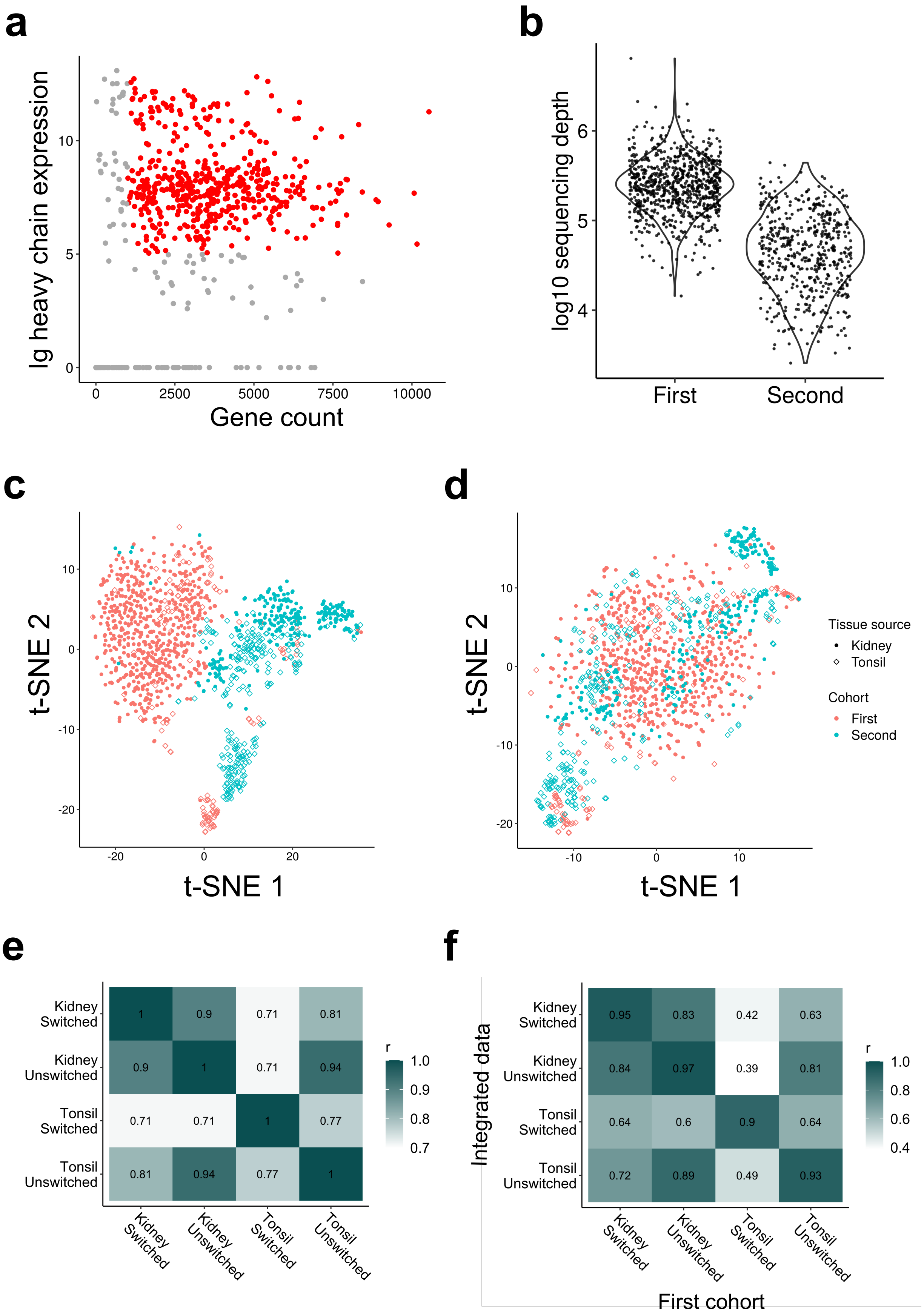
a
Ig heavy chain expression
Gene count
0
2500
5000
7500
10000
5
10
b
log10 sequencing depth
First
Second
4
5
6
c
t-SNE 2
t-SNE 1
-20
0
20
-10
10
d
t-SNE 2
t-SNE 1
-10
0
10
-20
Tissue source
Kidney
Tonsil
Cohort
First
Second
e
Kidney Switched
Kidney Unswitched
Tonsil Switched
Tonsil Unswitched
1
0.9
0.71
0.81
0.9
1
0.71
0.94
0.71
0.71
1
0.77
0.81
0.94
0.77
1
r
1.0
0.9
0.8
0.7
f
Integrated data
First cohort
Kidney Switched
Kidney Unswitched
Tonsil Switched
Tonsil Unswitched
0.95
0.83
0.42
0.63
0.84
0.97
0.39
0.81
0.64
0.6
0.9
0.64
0.72
0.89
0.49
0.93
r
1.0
0.8
0.6
0.4

Supplementary Figure 4. QC of the second cohort and data integration.

(**a**) A scatter plot showing detected gene count and Ig Heavy chain gene expression on the x and y axis respectively. Cells which passed QC are colored in red. (**b**) A violin plot showing sequencing depth of cells which passed QC in each cohort. (**c** and **d**) t-SNE plots made from the data normalized by log2 cpm without data integration (c) or the data normalized by SCTransform and integrated by ComBat (d). Color and shape respectively indicate cohorts and tissue source. (**e** and **f**) Pearson correlation of gene expression of B cell populations in the integrated data. Tested within the second cohort dataset for 1,000 most variant genes (e) or against the first cohort for 736 genes which were within top 1,000 high-variant genes in both datasets (f).

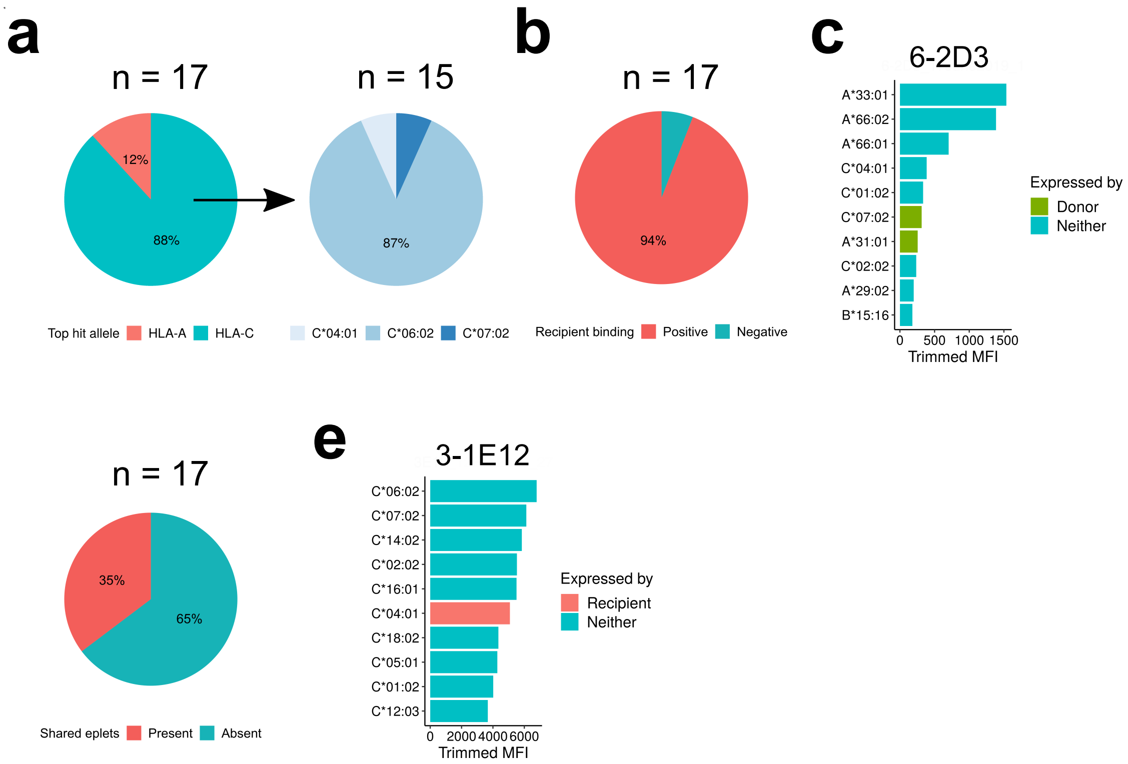

Supplementary Figure 5. SAB assay analysis.

(**a**) Pie charts showing top-hit alleles in the SAB assay. 17 antibodies with trimmed MFI ≥ 1,000 were analyzed. (**b**) A ratio of antibodies that had a recipient-expressed antigen among top-10 hits. (**c**) Trimmed MFI of 6-2D3. Donor-expressed antigens are colored in green. (**d**) A ratio of antibodies whose top-10 hits shared eplets. (**e**) Trimmed MFI of a representative antibody 3-1E12 with a shared eplet. A recipient-expressed antigen is colored in red.

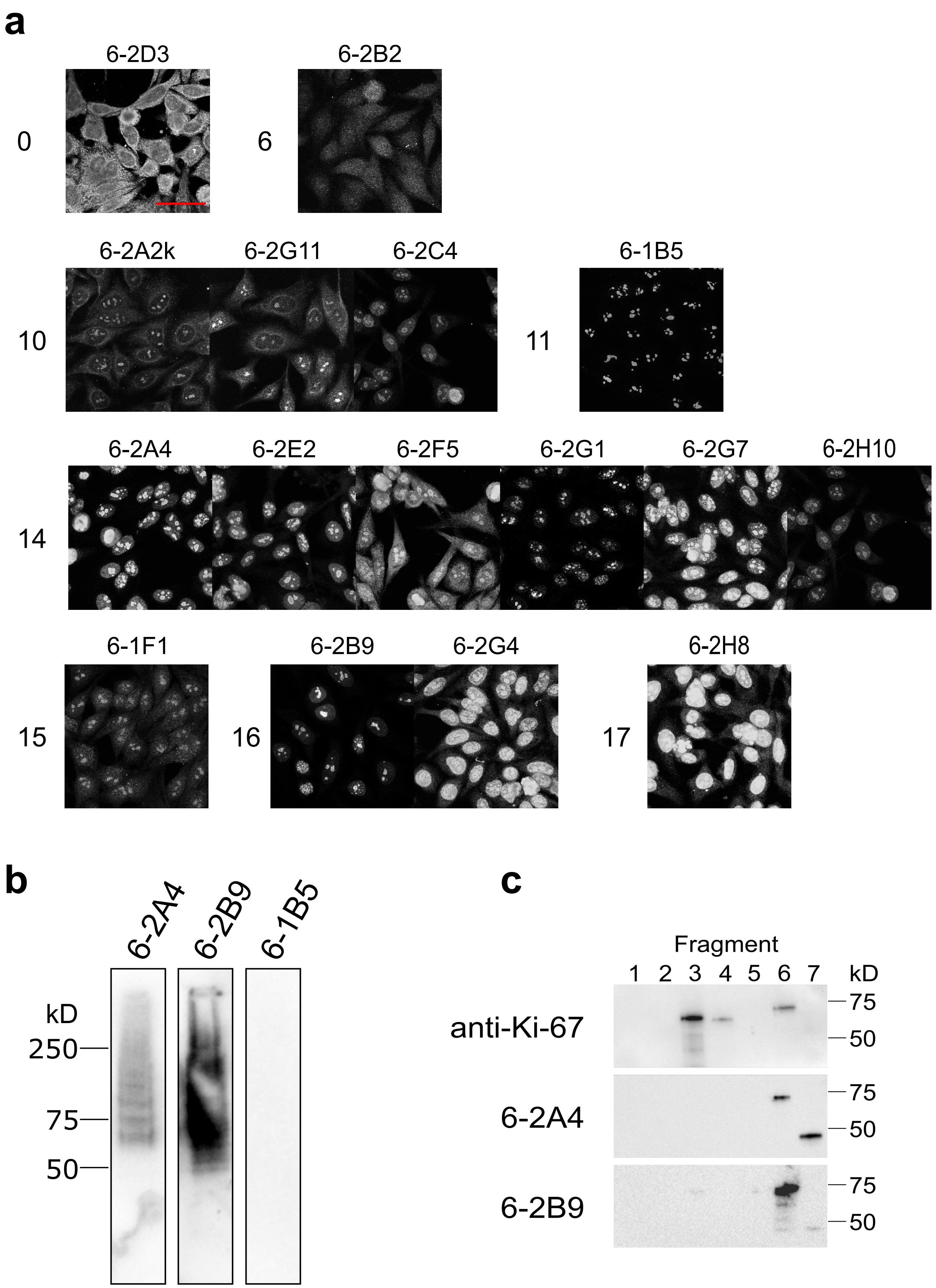
a
6-2D3
6-2B2
0
6
6-2A2k
6-2G11
6-2C4
6-1B5
10
11
6-2A4
6-2E2
6-2F5
6-2G1
6-2G7
6-2H10
14
6-1F1
6-2B9
6-2G4
6-2H8
15
16
17
b
6-2A4
6-2B9
6-1B5
kD
250
75
50
c
Fragment
1 2 3 4 5 6 7
kD
anti-Ki-67
6-2A4
6-2B9
75
50
75
50
75
50

Supplementary Figure 6. Autoreactivity of antibodies expressed by plasma cells in patient 6. (**a**) HEp-2 staining images of the 16 positive antibodies cloned from patient 6 plasma cells. Antibodies of the same clonal family are grouped together. Numbers on the left side of the images indicate their clonal family number as in Figure 6C. The scale bar indicates 50 μm. (**b**) Western blot on HEp-2 nuclear lysates using the three antibodies (6-2A4, 6-2B9, 6-1B5). (**c**) Western blot on E. coli lysates expressing Ki-67 fragments. Each fragment contains these amino acids: fragment 1, aa 1-526; fragment 2, aa 509-1009; fragment 3, aa 994-1489; fragment 4, aa1476-1976; fragment 5, aa1963-2459; fragment 6, aa2446-2940; and fragment 7, aa2927-aa3256. Experiments for (a-c) have been repeated twice and a representative image is shown. Uncropped western blot images are provided in the Source Data file.
