# Supplementary material for: Innate-like self-reactive B cells infiltrate human renal allografts during transplant rejection
Source: Nat Commun. 2021 Jul 16;12:4372. doi: 10.1038/s41467-021-24615-6 (PMC8285506; doi:10.1038/s41467-021-24615-6)
Supplement: Supplementary file 3 — Description of Additional Supplementary Files [file 41467_2021_24615_MOESM3_ESM.pdf]

**Description of Additional Supplementary Files**

File Name: Supplementary Data 1
Description: **A list of 2855 DEG.** Gene symbols are shown with their clusters identified in Figure 2d. Columns "comparison1-4" show which category expressed the genes significantly higher in each differential expression test.

File Name: Supplementary Data 2
Description: **A list of Ahnak and AHNAK-covariant genes.** Gene symbols of Ahnak-covariant mouse genes and their human orthologs (*AHNAK*-covariant genes) are shown.

File Name: Supplementary Data 3
Description: **A list of recombinantly expressed antibodies.** Information of heavy and light chain genes and assay results are shown.

File Name: Supplementary Data 4
Description: **Eplet analysis of SAB assay-positive antibodies.** Top-10 hit antigens, trimmed mean MFI, and eplets present in the antigens are shown for each antibody. Eplets shared among all top-10 hits are colored in red.

File Name: Supplementary Data 5
Description: **DNA sequences of primers and Ki-67 fragments.** Sequences of cloning primers and purchased DNA encoding Ki-67 fragments.
