# Supplementary material for: Innate-like self-reactive B cells infiltrate human renal allografts during transplant rejection
Source: Nat Commun. 2021 Jul 16;12:4372. doi: 10.1038/s41467-021-24615-6 (PMC8285506; doi:10.1038/s41467-021-24615-6)
Supplement: Supplementary file 9 — Reporting Summary [file 41467_2021_24615_MOESM9_ESM.pdf]

nature research

Corresponding author(s): Marcus R Clark

Last updated by author(s): 06/09/2021

Reporting Summary

Nature Research wishes to improve the reproducibility of the work that we publish. This form provides structure for consistency and transparency in reporting. For further information on Nature Research policies, see our Editorial Policies and the Editorial Policy Checklist.

Statistics

For all statistical analyses, confirm that the following items are present in the figure legend, table legend, main text, or Methods section.

n/a	Confirmed	
☐	☒	The exact sample size (*n*) for each experimental group/condition, given as a discrete number and unit of measurement
☐	☒	A statement on whether measurements were taken from distinct samples or whether the same sample was measured repeatedly
☐	☒	The statistical test(s) used AND whether they are one- or two-sided<br>*Only common tests should be described solely by name; describe more complex techniques in the Methods section.*
☐	☒	A description of all covariates tested
☐	☒	A description of any assumptions or corrections, such as tests of normality and adjustment for multiple comparisons
☐	☒	A full description of the statistical parameters including central tendency (e.g. means) or other basic estimates (e.g. regression coefficient) AND variation (e.g. standard deviation) or associated estimates of uncertainty (e.g. confidence intervals)
☐	☒	For null hypothesis testing, the test statistic (e.g. *F*, *t*, *r*) with confidence intervals, effect sizes, degrees of freedom and *P* value noted<br>*Give P values as exact values whenever suitable.*
☒	☐	For Bayesian analysis, information on the choice of priors and Markov chain Monte Carlo settings
☒	☐	For hierarchical and complex designs, identification of the appropriate level for tests and full reporting of outcomes
☐	☒	Estimates of effect sizes (e.g. Cohen's *d*, Pearson's *r*), indicating how they were calculated

Our web collection on statistics for biologists contains articles on many of the points above.

Software and code

Policy information about availability of computer code

Data collection	FACSDiva 8.0.1 was used for flow cytometry and cell sorting.

Data analysis	FlowJo 10.7.1<br>ImageJ 2.0.0<br>MaxQuant 1.5.2.8<br>Perseus 1.6.7<br>R 3.6.3<br>Python 3.7.1<br>RepeatMasker 4.1.0<br>kallisto 0.46.1<br>R clan packages:<br>Rtsne 0.15<br>BioConductor packages:<br>edgeR 3.26.8<br>RUVSeq 1.16.1<br>sva 3.32.1<br>clusterProfiler 3.12.0<br>org.Hs.eg.db 3.8.2<br>Seurat 3.1.1<br><br>All the code written to analyze and visualize data using publicly available programs is available at https://github.com/yutasano0121/Asano_NC_2021.

For manuscripts utilizing custom algorithms or software that are central to the research but not yet described in published literature, software must be made available to editors and reviewers. We strongly encourage code deposition in a community repository (e.g. GitHub). See the Nature Research guidelines for submitting code & software for further information.

Data

Policy information about availability of data

All manuscripts must include a data availability statement. This statement should provide the following information, where applicable:

- Accession codes, unique identifiers, or web links for publicly available datasets
- A list of figures that have associated raw data
- A description of any restrictions on data availability

All the data and code used in this paper will be available on request. Sequencing and mass spectrometry data have been deposited to the GEO (GSE162809) and PRIDE Archive (PXD022989) respectively.

Field-specific reporting

Please select the one below that is the best fit for your research. If you are not sure, read the appropriate sections before making your selection.

- [x] Life sciences
- [ ] Behavioural & social sciences
- [ ] Ecological, evolutionary & environmental sciences

For a reference copy of the document with all sections, see nature.com/documents/nr-reporting-summary-flat.pdf

Life sciences study design

All studies must disclose on these points even when the disclosure is negative.

Sample size	No statistical calculations were performed to determine sample sizes. Each data category had at least three samples.
Data exclusions	No data were excluded from the analyses.
Replication	scRNA-seq was performed in two separate cohorts. For antibody analysis, technical replicates were performed, and results were successfully repeated twice.
Randomization	For RNA-seq and microarray analysis, category of data points was determined by parameters indicated in the manuscript and figure legends (e.g. tissue sources, or Ig class-switched states). For recombinantly expressed antibodies, class-switched antibodies were preferentially selected so that more mutated antibodies would be studied. Within each Ig isotype, antibodies were randomly selected for expression.
Blinding	For scRNA-seq, library preparation and sequencing was done by a researcher without clinical information on samples. For HLA-binding assay in Figure 6, IP-mass spectrometry in Figure 7, and tissue staining in Figure 8, experiments were performed by researchers who did not know the origin of antibodies. For other staining assay, initial testing was performed without blinding, and positive staining was repeated by another researcher.

Reporting for specific materials, systems and methods

We require information from authors about some types of materials, experimental systems and methods used in many studies. Here, indicate whether each material, system or method listed is relevant to your study. If you are not sure if a list item applies to your research, read the appropriate section before selecting a response.

Materials & experimental systems

n/a	Involved in the study	
☐	☒	Antibodies
☐	☒	Eukaryotic cell lines
☒	☐	Palaeontology and archaeology
☒	☐	Animals and other organisms
☐	☒	Human research participants
☒	☐	Clinical data
☒	☐	Dual use research of concern

Methods

n/a	Involved in the study	
☒	☐	ChIP-seq
☐	☒	Flow cytometry
☒	☐	MRI-based neuroimaging

Antibodies

Antibodies used

Antibodies used are listed below [antibody name, manufacturer, clone ID, catalog ID, lot ID]
Flow Cytometory:
PE-CD19, ThermoFisher Scientific, SJ25C1, 12-0198-42, E13128-107
APC-CD38, BD Biosciences, HIT2, 555462, 1705405A
PE-Cy7-CD45, ThermoFisher Scientific, HI30, 25-0459-42, 5254739

Tissue Staining:
Rat anti-CD19, ThermoFisher Scientific, 6OMP31, 14-0194-82, 4313386
Rabbit anti-CD19, Abcam, EPR5906, ab134114, GR214860-11
Rabbit anti-AHNAK, Proteintech, Polyclonal, 16637-1-AP, 00008028
Mouse anti-IL15, Abcam, N/A, ab55276, GR3175423-6
Goat anti-IL15RA, ThermoFisher Scientific, Polyclonal, PA5-46991, TK2666477
Rabbit anti-Ki-67 abcam, EPR3610, ab92742, GR213055-51
Rat anti-FLAG, BioLegend, L5, 637301, B227795
Alexa Fluor 488 donkey anti-rat IgG, ThermoFisher Scientific, Polyclonal, A21208, 1932496
Alexa Fluor 488 donkey anti-rabbit IgG, ThermoFisher Scientific, Polyclonal, A21206, 1981155
Alexa Fluor 594 donkey anti-mouse IgG, ThermoFisher Scientific, Polyclonal, A21203, 1722995
Alexa Fluor 647 donkey anti-rabbit IgG, ThermoFisher Scientific, Polyclonal, A31573, 2083195
Alexa Fluor 647 Plus donkey anti-goat IgG, ThermoFisher Scientific, Polyclonal, A32849, TF271044

Western Blot:
HRP Goat Anti-Human IgG Fc, ThermoFisher Scientific, Polyclonal, 31413, JB11218310

HEp-2 staining
FITC anti-human IgG, iNova Diagnostics, Polyclonal, 426950 (included in NOVA Lite HEp-2 ANA kit), 051381

Validation

Commercial antibodies have been validated by manufacturers based on specific assays, such as side-by-side comparison with an isotype control antibody on flow cytometry, western blot comparing cell lines with or without target expression, and staining of multiple tissues with a known expression pattern of target proteins. Staining results of antibodies cloned from primary B cells were validated by technical replicates and/or multiple experimental systems (i.e. western blot, tissue staining, and mass-spec).

Eukaryotic cell lines

Policy information about cell lines

Cell line source(s)	HEK293 (ATCC, CRL-1573) and HEp-2 (ATCC, CCL-23).
Authentication	Cells were not authenticated after their purchase.
Mycoplasma contamination	Not tested.
Commonly misidentified lines (See ICLAC register)	Hek293 and HEp-2 cells were obtained from ATCC and cultured in a laboratory which did not have HeLa cells that are reported as a contaminating cell line according to ICLAC.

Human research participants

Policy information about studies involving human research participants

Population characteristics	Diagnostic renal biopsies from eight patients with renal allograft rejection, as well as seven tonsillectomy samples were collected. Clinical information of the allograft patients are listed in Supplementary Table 1. Tonsil donors have been de-identified.
Recruitment	Participants were recruited according to protocols approved by the internal review board at the University of Chicago. Informed consent was obtained from patients with for-cause biopsies. Then, biopsies from patients with antibody-mediated rejection evidenced by clinical histology were subjected to sequencing. Patients were not filtered by ethnicity, gender, age, or other clinical characteristics.
Ethics oversight	Institutional Review Board at the University of Chicago

Note that full information on the approval of the study protocol must also be provided in the manuscript.

Flow Cytometry

Plots

Confirm that:

- [x] The axis labels state the marker and fluorochrome used (e.g. CD4-FITC).
- [x] The axis scales are clearly visible. Include numbers along axes only for bottom left plot of group (a 'group' is an analysis of identical markers).
- [x] All plots are contour plots with outliers or pseudocolor plots.
- [x] A numerical value for number of cells or percentage (with statistics) is provided.

Methodology

Sample preparation	Cells were isolated from minced patient biopsies with Liberase-TL. Single-cell suspension was washed and stained in 2% BSA-PBS and analyzed and sorted with a flow cytometer.
Instrument	FACS Aria Fusion
Software	FACSDiva
Cell population abundance	Purity was not tested post-sort. However, cells without immunoglobulin gene expression based on sequencing data were removed from analyses, as indicated in the manuscript.
Gating strategy	Doublets were removed based on FSC-A/FSC-H gating. Live lymphocytes were selected by CD45-positive, DAPI-negative Calcein-positive and FSC-A/SSC-A gating. Within the selected population, CD19-positive CD38-positive cells were sorted.

- [x] Tick this box to confirm that a figure exemplifying the gating strategy is provided in the Supplementary Information.
